# CsTs, a C-type lectin receptor-like kinase, regulates the development trichome development and cuticle metabolism in cucumber (*Cucumis sativus*)

**DOI:** 10.1093/hr/uhae235

**Published:** 2024-08-14

**Authors:** Duo Lv, HaiFan Wen, Gang Wang, Juan Liu, ChunLi Guo, Jingxian Sun, Keyan Zhang, ChaoHan Li, Jiaqi You, Ming Pan, Huanle He, Run Cai, Junsong Pan

**Affiliations:** Shanghai Collaborative Innovation Center of Agri-Seeds / School of Agriculture and Biology, Shanghai JiaoTong University, Shanghai 200240, China; Shanghai Key Lab of Protected Horticultural Technology, Horticultural Research Institute, Shanghai Academy of Agricultural Sciences, Shanghai 201106, China; Shanghai Collaborative Innovation Center of Agri-Seeds / School of Agriculture and Biology, Shanghai JiaoTong University, Shanghai 200240, China; Shanghai Collaborative Innovation Center of Agri-Seeds / School of Agriculture and Biology, Shanghai JiaoTong University, Shanghai 200240, China; Shanghai Collaborative Innovation Center of Agri-Seeds / School of Agriculture and Biology, Shanghai JiaoTong University, Shanghai 200240, China; Shanghai Collaborative Innovation Center of Agri-Seeds / School of Agriculture and Biology, Shanghai JiaoTong University, Shanghai 200240, China; Shanghai Collaborative Innovation Center of Agri-Seeds / School of Agriculture and Biology, Shanghai JiaoTong University, Shanghai 200240, China; Shanghai Collaborative Innovation Center of Agri-Seeds / School of Agriculture and Biology, Shanghai JiaoTong University, Shanghai 200240, China; Shanghai Key Lab of Protected Horticultural Technology, Horticultural Research Institute, Shanghai Academy of Agricultural Sciences, Shanghai 201106, China; Shanghai Key Lab of Protected Horticultural Technology, Horticultural Research Institute, Shanghai Academy of Agricultural Sciences, Shanghai 201106, China; Shanghai Key Lab of Protected Horticultural Technology, Horticultural Research Institute, Shanghai Academy of Agricultural Sciences, Shanghai 201106, China; Shanghai Agricultural Technology Extension and Service Center, Shanghai 201100, China; Shanghai Collaborative Innovation Center of Agri-Seeds / School of Agriculture and Biology, Shanghai JiaoTong University, Shanghai 200240, China; Shanghai Collaborative Innovation Center of Agri-Seeds / School of Agriculture and Biology, Shanghai JiaoTong University, Shanghai 200240, China; Shanghai Collaborative Innovation Center of Agri-Seeds / School of Agriculture and Biology, Shanghai JiaoTong University, Shanghai 200240, China

## Abstract

Cucumber (*Cucumis sativus*) fruit spines are a classic material for researching the development of multicellular trichomes. Some key genes that influence trichome development have been confirmed to be associated with cuticle biosynthesis and secondary metabolism. However, the biological mechanisms underlying trichome development, cuticle biosynthesis, and secondary metabolism in cucumber remain poorly understood. *CsTs*, a C-type lectin receptor-like kinase gene, reportedly causes a tender trichome phenotype in cucumber when it mutates. In this study, the role of *CsTs* in cucumber fruit spines morphogenesis was confirmed using gene editing technology. Sectioning and cell wall component detection were used to analyse the main reason of tender fruit spines in the *ts* mutant. Subsequently, transcriptome data and a series of molecular biology experiments were used to further investigate the relationship between *CsTs* and cytoskeletal homeostasis in cucumber. *CsTs* overexpression partially compensated for the abnormal trichome phenotype of an *Arabidopsis* homolog mutant*.* Genetic hybridization and metabolic analysis indicated that *CsTs* and *CsMict* can affect trichome development and cuticle biosynthesis in the same pathway. Our findings provide important background information for further researching on the molecular mechanism underlying cucumber trichome development and contribute to understanding the biological function of C-type lectin receptor-like kinases.

## Introduction

Trichomes, protective tissues of the above-ground part of plants, are widely distributed on fruits, stems and leaves, and are pivotal in resisting various biological and abiotic stresses. In some plants, trichomes can also affect appearance and commercial value. Trichomes are divided into several types, such as glandular and non-glandular, multi- and unicellular, and branched and unbranched. Trichome are a specialized tissue of the epidermal cells, and because of their ease of observation [1, 2], they are generally considered as a classic model for researching cell proliferation and differentiation. Moreover, some genes that play important roles in the regulatory system of trichome development have also been shown to be closely related to secondary metabolism and cuticle metabolisms in plants [3–5].

Trichomes on *Arabidopsis* leaves are unicellular, multibranched, and non-glandular [6], and their morphogenetic process relies on a complex gene regulation network. A series of transcription factors regulate the initiation of *Arabidopsis* trichomes by competing and interacting with each other [7–11]. Moreover, cytoskeleton homeostasis plays a pivotal role in trichomes morphogenesis [12, 13]. Generally, actin primarily influences trichome cell shape, while microtubules typically affect the number of trichome branches [14–16]. A Kinesin-like calmodulin-binding protein (KCBP/ZWICHEL) can interact with F-actin and microtubules to influence trichome morphogenesis in *Arabidopsis* [17]. In *kcbp* mutants, trichomes exhibit a short stalk and only one or two branches [18]. Moreover, KCBP can interact with ANGUSTIFOLIA and TSC1, both of which play roles in regulating microtubule stability and trichome branching [19]. Cucumber has both non-glandular (Type I) and glandular (Type II) multicellular trichomes. Type I trichomes are usually visible and have distinct structures (stalk and base), especially on the fruit, and are often referred to as ‘fruit spines’ [20–22]. Unlike in *Arabidopsis*, a limited number of genes related to regulating cucumber trichome development have been identified. For example, *trichome-less* (*tril*) and *glabrous 3* (*gl3*) mutants (two different mutated forms of *Csa6G514870*, an HD-ZIP transcription factor gene) result in a glabrous appearance on stems, leaves, tendrils, and fruits [23, 24]. Another HD-ZIP transcription factor gene, *Csa3G748220*, has two different mutated forms, with the 2649-bp fragment deletion (*micro-trichome*/*tiny branched hair*/*glabrous 1*, *mict*/*tbh*/*csgl1*) causing a micro-trichome phenotype [25]. A single-nucleotide polymorphism (SNP) in *Csa3G748220* (*no pyramid-shaped head trichome*, *nps, csmict-L130F*) can lead to malformed trichomes [26]. Recently, *Csa3G748220* has also been reported to be involved in cuticle biosynthesis and secondary metabolism [5]. The *ts* (*tender spines*) mutant exhibits a trichome phenotype similar to that of *nps*. Based on the transcriptome data, the candidate gene of *ts* locus has been demonstrated to regulate type I trichome morphogenesis by influencing cytoskeletal homeostasis and auxin polar transport [27, 28]. In a previous study [27], *Csa1G056960* was mapped as a candidate gene of the *ts* locus. *Csa1G056960* encodes a C-type lectin receptor-like kinase (LecRLK) that belongs to the LecRLK gene family in plants. The LecRLK gene family is divided into three subfamilies based on the extracellular domain: G-, L-, and C-type LecRLK [29]. Unlike those on the other two subfamilies, studies on C-type *LecRLK* genes are limited, and their role remains unclear. In most plants, only one or two members of C-type *LecRLK* genes have been identified [30–32].

In this study, we explored the biological function of *Csa1G056960* using a series of molecular and cell biology experiments. These findings can provide important background information for future research on the molecular mechanism underlying trichome development and cuticle metabolism in plant.

## Results

### Functional validation of *Csa1G056960* at the *Ts* locus

In the previous study [27], a candidate gene, *CsTs*, that influences the development of fruit spines and trichomes was mapped in a 109.7-kb region on chromosome (Chr) 1. In this region, only *Csa1G056960* has an SNP in the second intron in the mutant (*ts*).

**Figure 1:**
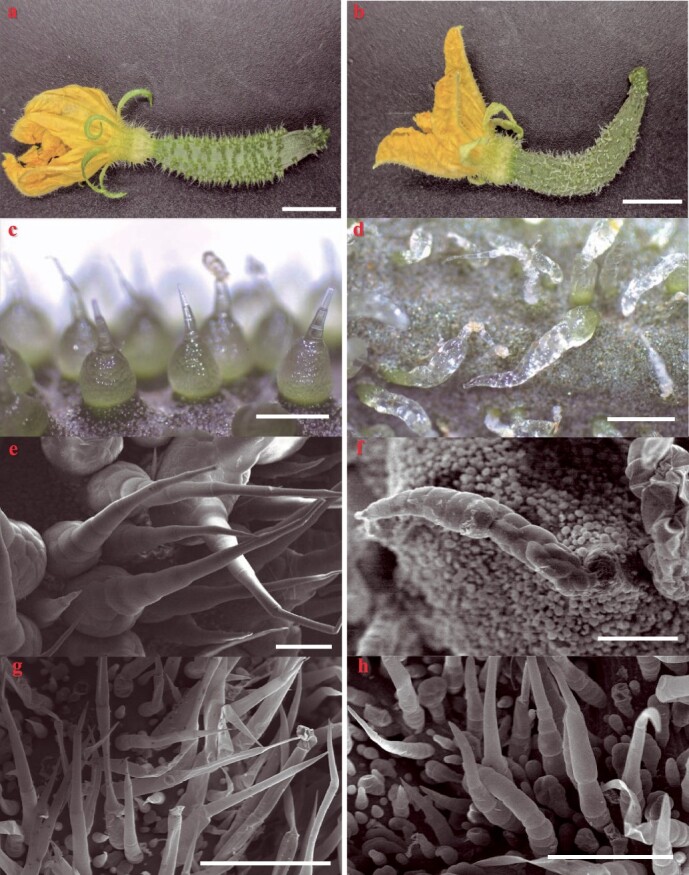
Functional verification of *CsTs* in cucumber. **a**, **b** The fruit of wild-type and *CsTs^CR^* cucumber. Scale bars = 1 cm. **c**, **d** The fruit spines of wild-type and *CsTs^CR^* cucumber. Scale bars = 1 mm. **e**, **f** The fruit spines of wild-type and *CsTs^CR^* cucumber. Scale bars = 250 μM. **g**, **h** The trichomes of wild-type and *CsTs^CR^* cucumber. Scale bars = 250 μM.

In the present study, to confirm that the role of *CsTs* was related to the morphogenesis of fruit spines and trichomes, CRISPR/Cas9 gene editing technology was used to generate *CsTs* mutants. Two homozygous knockout mutants harboring 1-bp (*CsTs^CR^#1*) or 10-bp deletions (*CsTS^CR^#2*) at the first single-guide RNA (sgRNA) target site were obtained. Both mutations led to a premature stop codon and generated a truncated protein (Fig. S1, see online supplementary material). Scanning electron microscopy (SEM) showed that the fruit spines of the two *CsTs^CR^* knockout lines were similar to those of the *ts* mutant. The structural differences between the stalk and base of the fruit spines disappeared (Fig. 1); the fruit spines of the *CsTS^CR^* lines were not erect but lay prostrate on the pericarp. Based on these results, we confirmed that the loss-of-function of *Csa1G056960* was the reason for fruit spine deformation in the *ts* mutant.

### A SNP mutation causes a localization change in cellular csts protein

**Figure 2:**
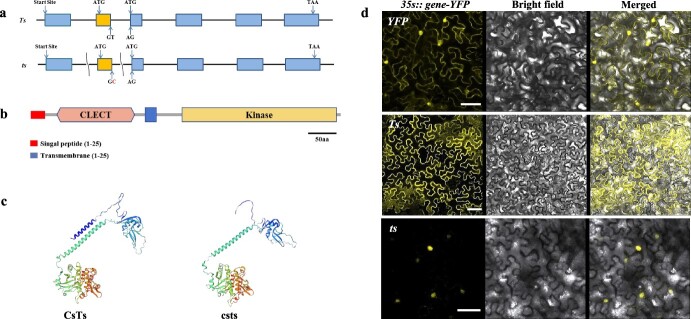
Effects of *csts* gene and protein mutation. **a** Gene structure of *CsTs* and *csts*, a single-nucleotide polymorphism mutation (T to C) in the second exon. **b** The conserved functional domain in CsTs protein. **c** 3D protein structure prediction of CsTs and csts proteins. **d** Subcellular localization of *CsTs-YFP* and *csts-YFP* fusion proteins in tobacco leaves. Scale bars = 50 μM.

The *CsTs* gene encodes a 557-amino-acid C-type LeRLK containing a signal peptide and lectin (extracellular), transmembrane (TM), and kinase (intracellular) domains. A cladogram based on the C-type LeRLK sequence alignment of six species revealed that the amino acid sequence identity ranged from 63 to 71%, and plants with the same type of trichomes were not clustered together (Fig. S2, see online supplementary material). Sequencing revealed a single-nucleotide mutation from T to C in the second intron (at the 5′ splicing site) in the *Csts* gene in the mutant. Compared with the wild-type (WT), this SNP causes the second exon deletion in the coding sequence (CDS) of the *csts* gene due to incorrect splicing [27] (Fig. 2a). Bioinformatics analysis showed that the second exon encodes the signaling peptide of CsTs protein and the SNP did not affect the protein structure except for signal peptide deletion (Fig. 2b and c), suggesting that cellular csts protein localization may change. Subcellular localization experiments showed that CsTs and csts proteins localized on the cell membrane and in the nucleus, respectively (Fig. 2d). RT-qPCR (Fig. S3a, see online supplementary material) and western blotting (Fig. S3b, see online supplementary material) of different tissues indicated no significant gene and protein expression differences between *CsTs* and *csts*.

### Mutation of *CsTs* gene causes a fruit spines malformation in mutant

**Figure 3:**
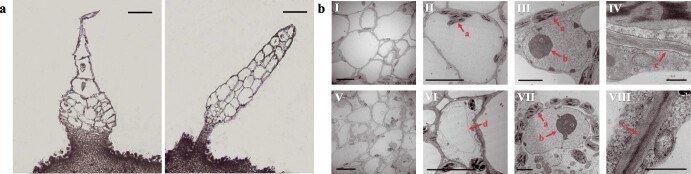
Structure differences of fruit spine cells between wild-type and *ts* cucumber mutant. **a** Paraffin sections of fruit spines: wild-type (left) and *ts* mutant (right). Scale bars = 200 μm. **b** TEM of fruit spines. (I–IV) TEM of wild-type; (V–VIII) TEM of *ts* mutant. a, Plastid; b, Nucleolus; c, Intercellular layer; d, Vacuole. (I, II, V, VI) Scale bars = 10 μm, (III, VII) Scale bars = 2.5 μm, (IV, VIII) Scale bars = 1 μm.

Paraffin sections showed the differentiation between the base and stalk of the WT fruit spines was apparent (Fig. 3a), whereas no obvious structural division in the fruit spines of the *ts* mutant was observed. However, the base structure enabled the tight connection of WT fruit spines to the cucumber epidermis, whereas *ts* mutant fruit spines were not firmly connected to the epidermis due to malformative development and the lack of a normal base structure.

Transmission electron microscopy (TEM Fig. 3b) revealed that the intercellular layer connections in *ts* mutant fruit spines were closer, and the numbers of cells per unit area and intracellular plastids were higher, indicating that WT fruit spine cells were more fully extended and the cell wall was relatively rigid.

Based on the above experimental data, we speculated that the ‘tender’ fruit spines phenotype in the *ts* mutant might be attributed not only to the change in cell division mode but also to cell wall composition. Cell wall composition analysis confirmed that the cellulose and lignin contents of WT fruit spines were higher, while those of pectin and hemicellulose were higher in *ts* mutant fruit spines (Table 1). In addition, the *ts* mutant had more fruit spines (705 ± 35) than those of the WT (557 ± 13.5, Table 2).

**Table 1:** Cell wall components of *ts* mutant and wild-type cucumber.

	**Genotype**			
**Component**	**WT**		**ts**	
	**Mean**	**SD**	**Mean**	**SD**
Pectin (mg/g)	32.99^a^	0.05	39.22	0.10
Cellulose (μg/mg)	39.81^a^	0.48	34.18	0.41
Hemicellulose (μg/mg)	25.06^a^	0.42	32.77	0.52
Lignin (μg/mg)	26.88^a^	1.38	16.27	1.71

a Significant changes between the wild-type and *ts* mutants (Student’s *t*-test, *P* < 0.05, *n* ≥ 4).

**Table 2:** Number of fruit spines in different cucumber genotypes.

**Genotype**	**Spines number of each fruit**
WT	557 ± 13.5^a^
ts	705 ± 35.0^b^
nps	1908 ± 97.5^c^

Lowercase letters above the number indicate signifcant differences according to Tukey’s multiple comparisons test (*P* < 0.05, *n* ≥ 4).

### Mutation of *CsTs* gene may disrupt the cytoskeletal system

**Figure 4:**
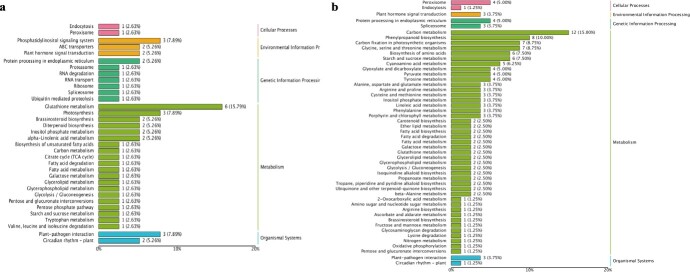
Analysis of transcriptome between wild-type and *ts* mutant of cucumber. **a** KEGG pathway analysis of the first group of DEGs. **b**KEGG pathway analysis of the second group of DEGs.

Fruit spine development can be divided into four stages, and an apparent change in the development pattern was observed after stage II in the *ts* mutant [28]. Transcriptome analysis in the previous study [28] indicated that *CsTs* could regulate the development of cucumber trichomes and fruit spines in stage III by influencing gene regulatory networks related to the cell cycle, auxin polar transport, and cytoskeletal system. TEM, cell wall component evaluation, and knockout mutant phenotype all indicated that *CsTs* still significantly influenced fruit spine development during stage IV. To further determine the global effects of *CsTs* on cucumber fruit spines development, we analysed the differentially expressed genes (DEGs) during stage IV (Table S2, see online supplementary material). We categorized the DEGs into two groups. The first group comprised genes (166 DEGs) that maintain the changes observed in stage III (Table S2, see online supplementary material). These DEGs represented genes that may be influenced by *CsTs* to mediate the overall development of fruit spines. Kyoto Encyclopedia of Genes and Genomes (KEGG) pathway analysis showed that DEGs were significantly enriched in plant hormone signal transduction, flavonoid metabolism, and fatty acid biosynthesis and metabolic pathways (Fig. 4a). The second group comprised DEGs whose expression levels did not change during stage III but changed in stage IV (413 DEGs) (Table S2, see online supplementary material). Their effect was delayed by *CsTs,* and they may be primarily involved in the development of the base structure of fruit spines.

Furthermore, KEGG pathways analysis showed that these DEGs were not only associated with hormone signal transduction, flavonoid metabolism, and fatty acid metabolism pathways but also with a broader range of secondary metabolism pathways (Fig. 4b). Another noteworthy point was that both DEG groups included some genes involved in cytoskeleton and auxin transport (Table S2, see online supplementary material), which are also associated with cell division and proliferation. For example, *PGP4* (*AT2G47000*, the homolog of *Csa2G074190*) can affect the directional growth of lateral roots in *Arabidopsis* by regulating the polar localization of auxin [33]; its expression level was downregulated by 6.75 folds in *ts* mutant. The homologous of *Csa7G372320* is *ATFIM2* (*AT5G48460*), which encodes a fimbrin protein in *Arabidopsis*. The fimbrin family primarily regulates the physical properties of the cytokeleton during the cell cycle by tightening and loosing actin and filaments [34]. The expression level of was *Csa7G372320* downregulated by 2.63 folds in *ts* mutant.

### Overexpression of *CsTs* can partially compensate for the abnormal trichome phenotype of its homologue mutant in *Arabidopsis*

Although the trichome structure in *Arabidopsis* differs from that in cucumber, the molecular regulatory mechanisms governing its development are interconnected. The morphogenesis of *Arabidopsis* trichomes is closely related to the cytoskeletal system [15], and the phenotype of the C-type *LecRLK* (*atts*, *AT1G52310*) mutant in *Arabidopsis* has not been reported. SEM revealed a significant increase in the number of trichome branches in the mutant *atts* compared with that in WT *Arabidopsis* (Col-0) (Fig. S4a and b, see online supplementary material), with the absence of two-branched trichomes, a notable increase in four-branched trichomes, and the appearance of five- and six-branched trichomes. To further investigate the biological function of *CsTs*, *35S::CsTs* was transferred to the *Arabidopsis* mutant *atts*. The number of trichome branches of the *oxCsTs* transgenic *atts* plants exhibited a significant reduction (Fig. S4c, see online supplementary material), with the absence of five-branched trichomes and a notable decrease in four-branched trichomes. These results indicated that the C-type *LecRLK* gene could affects the morphogenesis of multi and unicellular trichomes in different plants. 

### CsTs can interact with key factors related to cytoskeletal organization and auxin polar transport

**Figure 5:**
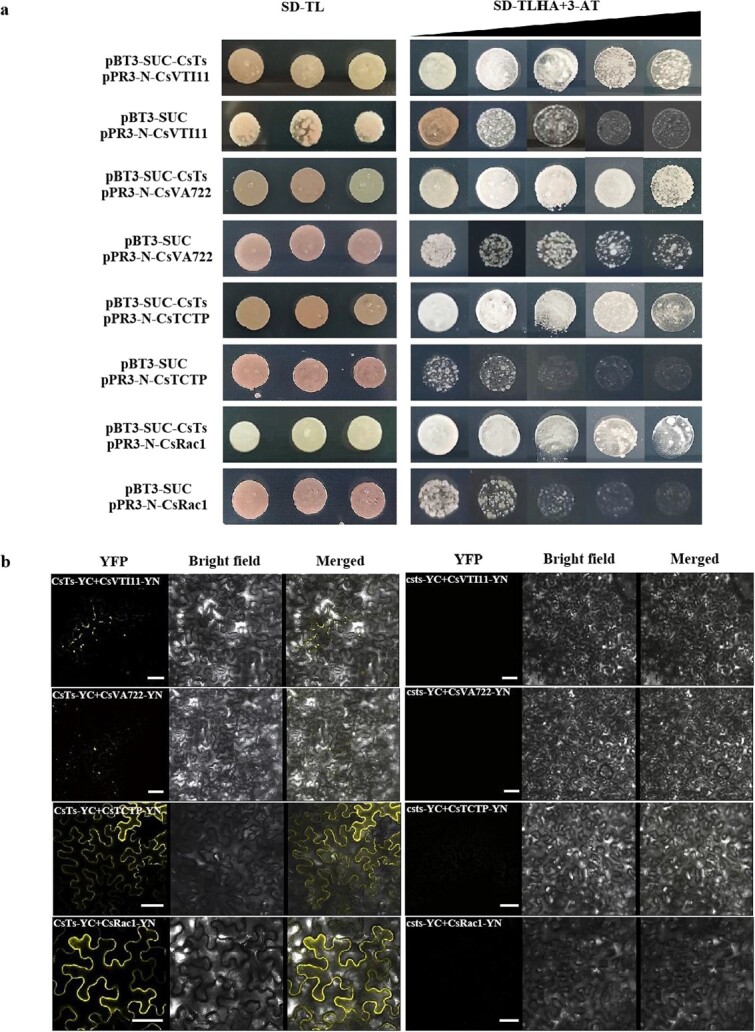
Interaction validation of screened proteins and CsTs of cucumber. **a** Yeast two-hybrid assays of the interactions between CsTs and screened proteins. **b** BiFC analysis between CsTs/csts and screened proteins. The concentration of 3-AT was 0, 1, 2.5, 5, and 7.5 mM. Scale bars = 50 μM.

To better simulate the CsTs protein expression pattern, a DUAL membrane system was selected to screen its interactors. Fifteen proteins that may interact with CsTs proteins were screened from the yeast two-hybrid library. Some of these proteins are closely related to cell proliferation and auxin polar transport (Table 3). Based on transcriptome data analysis, we selected six of these proteins for further one-to-one verification by bimolecular fluorescence complementation (BiFC) experiments (Fig. 5a). The results showed that four of them, Csa5G637740 (CsVTI11), Csa3G625650 (CsVA722), Csa3G154390 (CsTCTP), and Csa2G345950 (CsROP1), did indeed interact with CsTs, and the interaction sites were near the cell membrane. Concurrently, these four screened proteins also showed expression patterns similar to those of *CsTs* during the different developmental stages of fruit spines in WT cucumber (Table S3, see online supplementary material). In contrast, the four proteins did not interact with ts proteins *in vivo* (Fig. 5b) due to the abnormal csts location.

**Table 3:** Screened interaction proteins of CsTs from the yeast two-hybrid library.

**Gene ID**	**Function annotation**	**Localization**
Csa5G637740	Vesicle transport v-SNARE 11	CY
Csa4G365050	WAT1-related protein At4g08300	PM
Csa3G625650	Vesicle-associated membrane protein 722	CY
Csa2G200420	Auxin-responsive protein IAA14	NU
Csa3G154390	Translationally controlled tumor protein homolog	CY, NU, PM
Csa2G345950	RHO-related protein from plants	CY, PM

CY: cytoplasm, PM: Plasma membrane, NU: Nucleus.

The Csa3G625650 and Csa5G637740 homologs in *Arabidopsis* included VAMP722 (Target SNARE, t-SNARE) and VTI11 (Vacuolar SNARE, v-SNARE), respectively. The t-SNARE and v-SNARE of *Arabidopsis* can form complexes to regulate cell plate formation [35], ion channel transport [36], and gravitation by mediating the feedback between the auxin gradient and the cytoskeleton [37]. Csa3G154390 is a translationally controlled tumor protein (TCTP) that can bind Ca^2+^, microtubules, and Ras GTPase [38]. TCTP is associated with maintaining cytoskeletal stability, auxin transport, and the cell cycle, which can affect mitosis, especially the proliferation and growth of cancerous cells [39]. *Csa2G345950* encodes ROP1 protein, which belongs to the GTPases. In *Arabidopsis*, the role of ROP1 is related to cytoskeleton organization, establishment or maintenance of cell polarity, and cell shape regulation [40, 41]. It can interact with RIC3 and RIC4 to promote the proper targeting of exocytic vesicles [42].

### *CsTs* and *CsMict* were involved in the same pathway in regulating trichome development and cuticle metabolism

**Figure 6:**
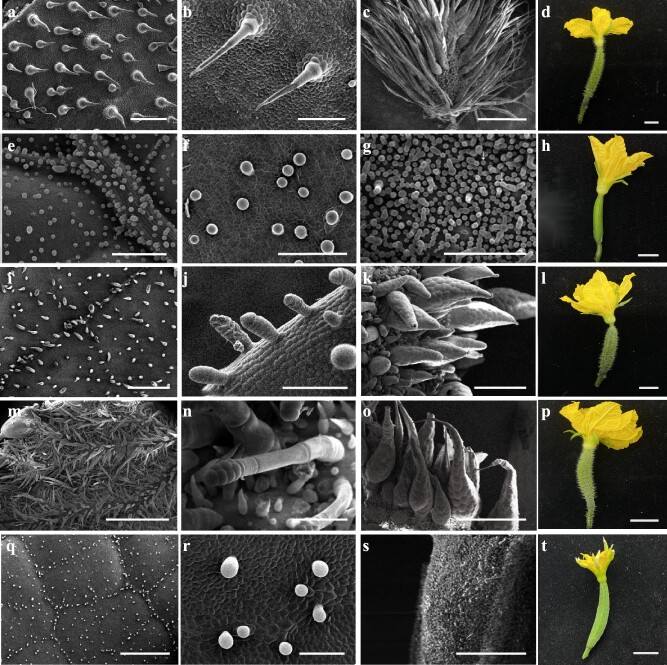
SEM of trichome and fruit spines from different types of cucumber. **a**, **b** Trichomes on the leaves of wild-type cucumber. **c**, **d** Wild-type spines. **e**, **f** Trichomes on the leaves of the *mict* mutant. **g**, **h**  *mict* mutant spines. **i**, **j** Trichomes on the leaves of the *nps* mutant. **k**, **l**  *nps* mutant spines. **m**, **n** Trichomes on the leaves of the *ts* mutant. **o**, **p**  *ts* mutant spines. **q**, **r** Trichomes on the leaves of the *nps*/*ts* mutant. **s**, **t**  *nps*/ *ts* mutant spines. (a, b, e, g) Scale bars = 200 μm, (f, j) Scale bars = 100 μm, (c, m, o) Scale bars = 1 mm, (i, k, n, q, r, s) Scale bars = 500 μm, (d, h, l, p, t) Scale bars = 1 cm.

A mutant of *CsMict* gene [26], *nps*, has a fruit spine morphology similar to that of *ts* mutants and *CsTs^CR^* knockout lines. In the present study, transcriptome data analysis demonstrated that the expression levels of *CsMict* were downregulated in *ts* mutants [28] (Table S2, see online supplementary material). Meanwhile, the number of fruit spines in *nps* and *ts* mutants significantly increased (Table 2). These data indicated that *CsMict* and *CsTs* could affect fruit spine development in the same biological pathway. To confirm this hypothesis, a *nps* and *ts* double mutant was constructed. SEM showed that the fruit spines of the double mutant had a phenotype similar to that of *mict*/*tbh*/*gl1*, wherein initial trichome and fruit spine development was inhibited (Fig. 6).

The study of *Csa3G748220* revealed that cuticle composition is dramatically altered in the *mict* mutant [5, 26]. To confirm that *CsTs* also affected cuticle biosynthesis, GC–MS analysis was performed to analyse leaf composition in *ts* and *nps* mutants (Table S3, see online supplementary material). Compared with that of the WT, the content of various cutin monomer components among the WT, *ts* and *nps* mutants did not significantly differ. On the other hand, the concentration of some types of long- or very long-chain fatty acids slightly increased in the *ts* and *nps* mutants compared with that in the WT. However, the wax content of the *ts* and *nps* mutants decreased to 81.33 and 64.22% of the WT, respectively, due to the significantly reduced leaf alcohol and alkane concentrations. These findings suggested that the loss-of-function of *CsMict* and *CsTs* affects the cuticle composition biosynthesis of cucumber leaves. At the same time, we also found that the influence of cuticle composition decreased in the order of *csmict* > *csmict-L130F* > *csts*.

Although the above data suggested that *CsTs* and *CsMict* were involved in the same pathway in regulating trichome/fruit spine development and specific metabolism in cucumber, BiFC experiments demonstrated no direct interaction between the CsTs and CsMict proteins (Fig. S5, see online supplementary material).

## Discussion

The trichomes on the aerial organs of cucumber are multicellular. To date, several genes related to the development of cucumber trichomes have been identified, including but not limited to *CsTril*, *CsMict*, and *CsNs* [22, 23, 25]. However, most of these genes are transcription factors, and their function is associated with initial trichome development. The recent discovery of the *ts* and *nps* mutant [26] has allowed further exploration of the molecular mechanisms underlying cucumber trichome development. In a previous study, *CsTs* was defined as a candidate gene that can affect the hardness of cucumber trichomes [27]. Nevertheless, the reason for the tender trichomes in the *ts* mutant was not thoroughly explored, and *CsTs* was not conclusively confirmed as the gene responsible for the mutant phenotype. *CsTs* is a plant-specific C-type *LecRLK* gene, and knowledge about the function of this type of gene is limited. Therefore, exploring the function of *CsTs* will not only enhance our understanding of the molecular mechanism involved in trichome development but will also help fill the gap in C-type *LecRLK* research. In the present study, CRISPR/Cas9 gene editing technology confirmed *CsTs* as a gene affecting the morphogenesis of cucumber trichomes and fruit spines, and its function was investigated by transcriptome analysis, cytology, genetics, and molecular biology experiments.

Here, we comprehensively analysed the main reasons for the tender fruit spine phenotype of the *ts* mutant and attributed it to changes in cell division and proliferation modes through sectioning and detection of cell wall components. First, the base of fruit spines became cylindrical instead of hemispherical in the *ts* mutant, especially at the bottom of the base, and the number of cells in contact with the pericarp significantly decreased. This results in an unstable attachment of the fruit spines to the epidermis. The twisted fruit spines of knockout mutants that lay flat on the pericarp further confirmed this hypothesis. Second, as the shape of the whole fruit spines in the *ts* mutant was deformed, more cells were squeezed into a similar-sized space, resulting in changes in the cell wall composition of the fruit spines to ensure better cell adhesion. Cellulose and hemicellulose content in the fruit spines of the *ts* mutant was significantly reduced. This was particularly evident under TEM, where the connections of the intercellular layer in *ts* mutant fruit spines were closer compared with those in the WT. Transcriptome data also indicated that some DEGs were closely associated with carbon and cell wall synthesis. These results suggest differences in fruit spine cell wall construction between the WT and *ts* mutant, resulting in *ts* mutant fruit spines that are tender to the touch.

The concentration gradient caused by unevenly distributed intracellular substances is the basis for cell polarity, and establishing and maintaining cell polarity primarily depend on cytoskeleton organization and vesicle transport [43]. The cytoskeletal system has been shown to be the main factor affecting the morphological formation of *Arabidopsis* trichomes. Once cytoskeletal organization is disrupted, the number of branches and density of *Arabidopsis* trichomes also change [44, 45]. Cucumber trichomes are multicellular, and the shape of their cells is asymmetrical, indicating active material transport during cucumber trichome development. Generally, C-type *LecRLK* gene comprises only one subfamily member in most plats [46]; to date, there have been no well-characterized research of their function. Our laboratory initially discovered that the C-type *LecRLK* gene could influence plant trichome development [27], and proposed that the *CsTs* gene may regulate trichome development by affecting the cytoskeletal system [28]. In the present study, we found more evidence to support this viewpoint. First, through yeast two-hybrid screening and BiFC, we identified CsTs-interacting proteins related to cell proliferation regulated by the cytoskeleton, and the mislocalization of csts protein suggested that it could not interact with these identified proteins. Second, we identified DEGs closely associated with the cytoskeletal system and auxin polar transport in the comparative transcriptome data from different stages of fruit spine development. At the same time, some genes related to Ca^2+^ transport also showed differential expression (Table S2, see online supplementary material), consistent with Yang *et al.* [47], which demonstrated that *CsTs* could affect Ca^2+^ concentration in fruit spines. One key mechanism by which the cytoskeletal system regulates cell polarity is modulating Ca^2+^ concentration near the membrane [48]. Several crucial genes determining the number and morphology of trichomes in *Arabidopsis* are also associated with Ca^2+^ binding [15]. Thirdly, only one C-type *LecRLK* gene is found in *Arabidopsis* [30], and its mutant exhibits a higher number of branches in its trichomes compared with that in Col-0, with *CsTs* overexpression partially rescuing this mutant phenotype. Moreover, evolutionary tree analysis of C-type LecRLK did not distinguish plants with different types of trichomes. This suggests that *CsTs* may be conservative in regulating trichome development and also suggests a relationship between the developmental mechanisms of uni- and multicellular trichomes to a certain extent. Based on the above findings, we summarized the regulatory mechanism of C-type LecRLK in plant trichomes as follows (Fig. S6, see online supplementary material): C-type LecRLK is localized on the cell membrane and interacts via intercellular signaling through its N-terminal extracellular domain. Subsequently, it transmits these signals through an intracellular kinase domain to regulate cell polarity. In multicellular trichomes, the CsTs protein ensures the orderly proliferation and differentiation of different structures, and in unicellular trichomes, it ensures that the cells extend 2–4 branches with a right angle.

To date, only a few key genes that regulate cucumber trichome development have been identified, and the relationship between some of them has been confirmed. For example, CsTTG1 can directly interact with CsMict [49], and *CsMYB649* directly regulates *CsTRY* [50]. Genetic analysis showed that *CsTril* has an epistatic effect on the *CsMict* and *CsNs* [22, 23]. *nps* is a mutant of *Mict*, which has a similar appearance of trichomes with the *ts* mutant. The *ts*/*nps* double mutant displayed a trichome appearance similar to that of the *mict* mutant, suggesting that *CsTs* was epistatic to *CsMict* in trichome development. However, the effect of *CsMict* on the morphogenesis of cucumber trichomes seems to be more pronounced than that of *CsTs* because *CsMict* affects not only the development of the stalk and the fruit spine base but also the apical cell shape. Some genes that related to trichome development have also been shown to play important roles in biological processes of secondary metabolism and cuticle metabolism in *Artemisia annua.* Several transcription factors have been confirmed that related to the artemisinin accumulation through regulating artemisinin biosynthesis and trichome development [3, 51, 52]: AaHD8 can affect the trichome initiation and leaf cuticle development by regulating the expression of multiple genes; and the trichomes in a hairless (*hl*) mutant of tomato show a malformed phenotype and reduced density, and accompanied by reductions in sesquiterpenes and polyphenols [53]. In our study, *CsTs* and *CsMict* similarly affected leaf cuticle metabolism regulation, but the effect of *CsTs* was inferior to that of *CsMict*, suggesting other factors affecting the regulatory pathway between *CsTs* and *CsMict*. *CsTs* has been shown to enhance aphid resistance in a recent study [47]. We also previously observed that aphids prefer to feed on *ts* mutants than on the WT in a greenhouse. We speculate that this feeding preference could be primarily due to a reduction in wax and secondary metabolites rather than due to changes in the cell wall thickness of trichomes.

In this study, *Csa1G056960* was confirmed at the *ts* locus using a *Ts* knockout mutant generated using CRISPR/Cas9. Its biological functions were explored using molecular and cell biology experiments. These results provide valuable molecular information for further studies on cucumber trichome development and cuticle metabolism, and contribute to understanding the biological function of C-type *LecRLK* genes in plants. However, due to the high difficulty of experimental techniques and limited reference research, the deep relationship between C-type *LecRLK* gene and the cytoskeleton system, as well as secondary metabolism requires more conclusive experimental data to be confirmed. For example, the cytoskeletal structure differences between *ts* mutants and wild-type, the effect of C-type *LecRLK*s mutations on the development of different types of trichomes, and whether phosphorylation is the main mode of interaction between CsTs proteins and several interactors. These questions will also be the focus of our future research.

## Materials and methods

### Plant materials

The North China type ‘NC072’ (wild-type, *Ts*) and its mutant ‘NC073’ (*ts*) were provided by Tianjin Derit Seeds Company Ltd (Tianjin, China). All cucumber plants in this study were grown at Shanghai Jiaotong University in 2019, 2020, and 2021 under natural photoperiodic conditions. For phenotypic analysis, at least three biological replicates were used.

### Cucumber transformation with CRISPR/Cas9 assay

To create CRISPR/Cas9 gene editing vectors, we designed two specific sgRNA target sites for *CsTs* by http://skl.scau.edu.cn/ [54]. A 626-bp fragment was amplified from the pCBC-DT1T2 vector template using primers that included sgRNA sequences, which was then inserted in the binary CRISPR/Cas9 vector pKSE401 [55]. The recombinant vector was transformed into *Agrobacterium tumefaciens* strain EHA105, and cucumber transformation was performed as described previously [56]. The genomic DNA of putative transgenic lines were extracted to identify knockouts. The primers used are listed in Table S1 (see online supplementary material). To ensure the accuracy of the phenotypic investigation, SEM was used to observe the fruit spines on cucumbers’ fruit on the flowering day.

### Subcellular localization

The CDSs of *CsTs* and *csts* were fused into the pHB-35S::X-YFP vector to construct the fusion proteins CsTs-YFP and csts-YFP. The transient expression of the fusion proteins in the epidermal cells of tobacco (*Nicotiana tabacum*) leaves was achieved according to the previous study [57]. After growing in the dark for 12 h and in light for 24 h at 26°C, a Leica TCS SP5 confocal fluorescence microscope (Leica, Wetzlar, Germany) was used to visualize the fluorescent signals.

### Bioinformatics tools and methods

The bioinformatics website SMART (http://smart.embl.de/) [58] was used to predict protein functional domains. Signal peptides were predicted using SignalP 5.0 (http://www.cbs.dtu.dk.services/SignalP) [59]. SWISS-MODEL (http://www.expasy.org/swissmod/SWISS-MODEL.html) was used to predict the 3D structure of proteins.

The sequences of CsTs proteins and their homologs in Arabidopsis (*Arabidopsis thaliana*, AT1G52310), tomato (*Solanum lycopersicum*, Solyc02g068370), cotton (*Gossypium hirsutum* L., GhA01G155200), rice (*Oryza sativa* L., Os01g01410), and *A. annua* L. were obtained from the NCBI database (https://www.ncbi.nlm.nih.gov/). The phylogenetic tree was constructed in MEGAX (v.10.1.8) using the neighbor-joining method.

### Western blotting

Proteins were separated by SDS-PAGE and transferred to a polyvinylidene difluoride membrane (0.45 μm; MilliporeSigma, Burlington, MA, USA). Thereafter, the polyvinylidene difluoride membrane was incubated with specific antibodies. An ECL kit (Cwbio, Beijing, China) was used to dye the membrane after incubation, after which a ChemiDoc imaging system (Bio-Rad Laboratories, Hercules, CA, USA) was used to detect the signal.

### Sample collection and RT-qPCR

Roots (4-week-old seedlings), cotyledons (4-week-old seedlings), true leaves (12-week-old seedlings), tendrils (12-week-old seedlings), female flowers (12-week-old seedlings), and cucumber fruits of different lengths (unfertilized; sepal, petal, and pistil removed) were collected from cucumber plants to investigate gene expression characteristics using RT-qPCR. RNA was extracted using a Plant Total RNA Isolation Kit (Sangon Biotech, Shanghai, China), and cDNA was prepared using MightyScript Plus First Strand cDNA Synthesis Master Mix (Sangon Biotech, Shanghai, China). RT-qPCR was conducted using FastStart Essential DNA Green Master Mix (Roche, Mannheim, Germany). The internal control for RT-qPCR was *CsActin3* (*Csa6G484600*). RT-qPCR was performed using a CFX Connection Real-Time System (Bio-Rad Laboratories, CA, USA). Three biological replicates were analysed for each treatment; each biological replicate contained three technical replicates. The data were analysed using the 2^−△△CT^ method [60]. The primers used are listed in Table S1 (see online supplementary material).

### Paraffin sections and scanning electron microscopy

Cucumber fruits were cut into 2–3-cm^2^ pieces, fixed in formalin-acetic acid-alcohol (FAA) [50% (v/v) ethanol, 5% (v/v) acetic acid, and 3.7% (v/v) formaldehyde], vacuumed for 30 min, and dehydrated using a gradient ethanol series [from 50 to 100% (v/v)] every 10 min.

For SEM, samples were pretreated using a Leica EM CPD300 desiccator and Leica EM SCD050 ion sputter and carbon coating unit (Leica, Wetzlar, Germany) and observed using an ALTO 1000 scanning electron microscope (Gatan, Inc., Pleasanton, CA, USA). For paraffin sectioning, samples were embedded in paraffin and sliced to a 10-μm size using a Leica RM 2126 Microtome (Leica, Wetzlar, Germany).

### Transmission electron microscopy

Flowering day cucumber fruit spines were fixed in 2.5% (w/v) glutaraldehyde, vacuumed for 20 min, and rinsed with 0.1 M phosphate buffer. Thereafter, 1% Hungry acid was used to post-fix the samples, which were then washed with 0.1 M phosphate buffer. Subsequently, the fruit spines were dehydrated using a gradient ethanol series [from 30 to 100% (v/v)] every 40 min. Finally, the fruit spines were embedded in Spurr’s resin, cut using a Leica UC6I microtome (Leica, Wetzlar, Germany), and imaged using a scanning transmission electron microscope (Tecnai G2 spirit Biotwin, Thermo Fisher, Waltham, MA,USA).

### Transcriptome data

Here, we used the transcriptome data from our previous study [28] (accession number: PRJNA660492).

### Yeast two-hybrid assay

A split-ubiquitin-based membrane yeast two-hybrid system (DUAL membrane System Biotech) assay was used to screen CsTs protein interactors. The construction and primary screening of the DUAL membrane System library were both completed by OE Biotech Co., Ltd (Shanghai, China).

The cDNA sequences (without the first 75 bp) of *CsTs* were fused into the bait vector pBT3-SUC, while those of *CsVA722*, *CsVTI11*, *CsTCTP,* and *CsROP1* were fused into the prey vector. Next, the recombinant bait and prey vectors were transformed into the specific yeast strain of the DUAL membrane System NMY51 and grown on SD media-Leu/−Trp and SD media-His/−Leu/−Trp/−Ade (with different concentrations of 3-AT). At least three independent experiments were performed, and the results of one representative experiment are shown. The primers used are listed in Table S1 (see online supplementary material).

### Bimolecular fluorescence complementation assay

The cDNA sequences of *CsTs*, *csts*, *CsVA722*, *CsVTI11*, *CsTCTP*, and *CsROP1* were fused with the pXY104 and pXY106 vectors. Tobacco leaves were used as a medium of expression as previously described [61]. After growing in the dark for 12 h and in light for 24 h at 26°C, a Leica confocal fluorescence microscope (TCS SP8, Leica, Wetzlar, Germany) was used to detect the fluorescence signals and obtain images. The primers used are listed in Table S1 (see online supplementary material).

### Cuticular wax and cutin chemical analysis

Fully expanded leaves (without trichomes) of the WT and *ts* and *nps* mutants were used to extract cuticular wax (at least three independent experiments were performed). For cutin analysis, fully expanded leaves were delipidated in a mixture of methanol-chloroform (1:1, v:v) for 2 weeks, after which the samples were dried and used for analysis as per the previously study [62].

### Genetic analysis of the relationship between *CsTs* and *CsMict*

F_1_ and F_2_ populations were established by crossing the homozygous *ts* mutant with the homozygous *nps* mutant. dCAPS markers (Table S1, see online supplementary material) designed for the SNPs of *CsTs* and *CsMict* were used to screen F_2_ populations, and sequencing was conducted to ensure the successful construction of a homozygous double mutant. To ensure the accuracy of the phenotypic investigation, SEM was used to observe the fruit spines on cucumber fruit on the day of flowering.

### T-DNA insertion mutant of C-type *LecRLK* identification and ectopic expression of *CsTs* in *Arabidopsis*

The C-type *LecRLK* gene (*AT1G52310*) T-DNA insertion mutant of *Arabidopsis* (*atts*) was purchased from AraShare (www.arashare.cn), and the identified primer was designed using T-DNA Primer Design (http://signal.salk.edu/tdnaprimers.2.html). The full-length CDS of *CsTs* and *csts* were cloned into the pHB-35S::X-YFP vector to generate overexpressing plants. The recombinant vector was transformed into *atts* using the floral dip method [50]. The transformed seeds were planted on 30 μg/mL hygromycin for primary screening and then re-screened by PCR using identification primers (Table S1, see online supplementary material).

### Statistical analyses

The data in this study are presented as mean ± standard deviation (SD) of biological replicates. Student’s *t*-test was performed to assess the statistical significance of treatment differences when only two individuals were compared. *P* values belong to one-way ANOVA (Tukey’s multiple comparisons test, α = 0.05). GraphPad Prism version 8.2.1 (GraphPad Software, La Jolla, CA, USA) was used for the statistical analyses. The statistical significance was considered as *P* < 0.05.

## Supplementary Material

Web_Material_uhae235

## Data Availability

All data generated or analysed during this study are included in this published article and its additional files.
