# Supplementary material for: CsTs, a C-type lectin receptor-like kinase, regulates the development trichome development and cuticle metabolism in cucumber (Cucumis sativus)
Source: Hortic Res. 2024 Aug 14;11(10):uhae235. doi: 10.1093/hr/uhae235 (PMC11489597; doi:10.1093/hr/uhae235)
Supplement: Web_Material_uhae235 [file web_material_uhae235.zip › Figuer S1.docx]

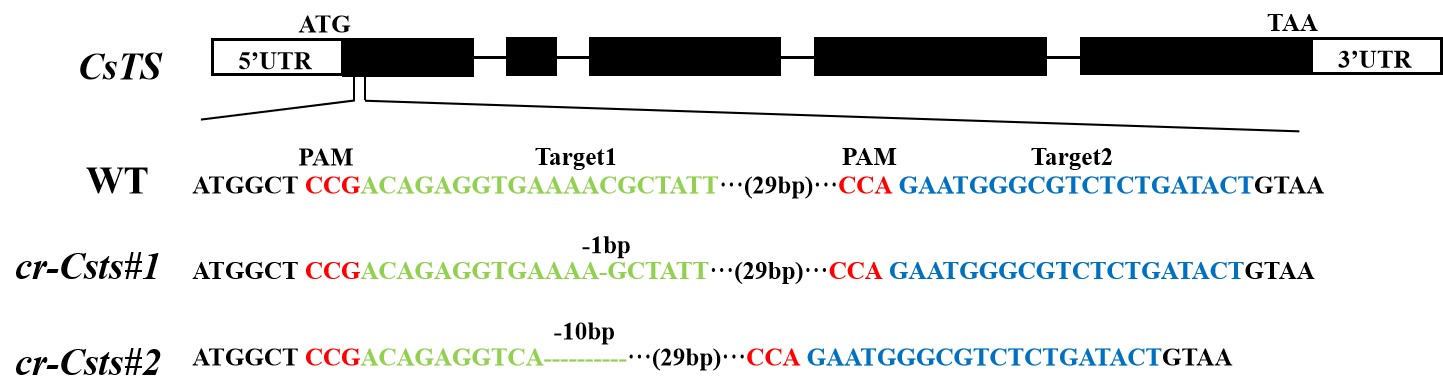


**Figure S1 Functional verification of *CsTs* in cucumber**

(A) The fruit spines observation of SEM to different type of cucumbers, Scale bars are 500μM;
(B) Schematic diagram showing the location of the sgRNA edit in *CsTS* genomic sequence and the two *CsTs^CR^* mutant alleles generated by CRISPR-Cas9
