# Supplementary material for: CsTs, a C-type lectin receptor-like kinase, regulates the development trichome development and cuticle metabolism in cucumber (Cucumis sativus)
Source: Hortic Res. 2024 Aug 14;11(10):uhae235. doi: 10.1093/hr/uhae235 (PMC11489597; doi:10.1093/hr/uhae235)
Supplement: Web_Material_uhae235 [file web_material_uhae235.zip › Figure S2.docx]

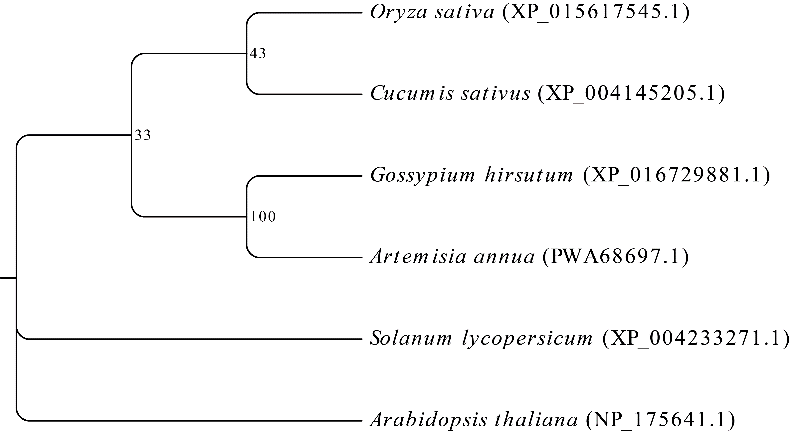


**Figure S2 The phylogenetic analysis of C-type LecRLK from different plants**

The value of the node represents bootstrap. The higher the value of bootstrap represents the closer the evolutionary relationship between species in the same branch.
