# Supplementary material for: CsTs, a C-type lectin receptor-like kinase, regulates the development trichome development and cuticle metabolism in cucumber (Cucumis sativus)
Source: Hortic Res. 2024 Aug 14;11(10):uhae235. doi: 10.1093/hr/uhae235 (PMC11489597; doi:10.1093/hr/uhae235)
Supplement: Web_Material_uhae235 [file web_material_uhae235.zip › Figure S3.docx]

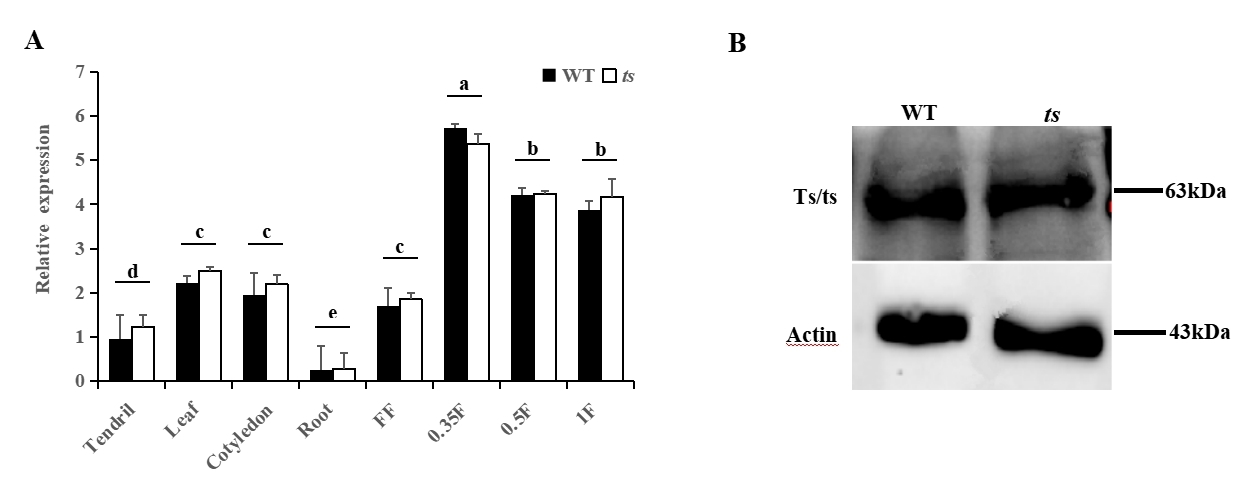


**Figure S3 The expression analysis of *CsTs* gene in wild type and *ts* mutant of cucumber**

(A) Relative expression level of *CsTs* gene in different organs of the wild-type (WT) and *ts* mutant (*ts*); (B) CsTs protein in WT and *ts* mutant.

**0.5F** Fruit of 0.5cm length (without sepal, petal, pistil and petiole), **1.0F** Fruit of 1.0cm length (without sepal, petal, pistil and petiole), **1.5F** Fruit of 1.5cm length (without sepal, petal, pistil and petiole), **FF** Female flower (without sepal, petal, pistil and petiole). Data are displayed as the ratio of expression to *CsActin* with three biological replicates. Lowercase letters above the bars indicate significant differences according to Tukey’s multiple comparisons test with *P*<0.05.
