# Supplementary material for: CsTs, a C-type lectin receptor-like kinase, regulates the development trichome development and cuticle metabolism in cucumber (Cucumis sativus)
Source: Hortic Res. 2024 Aug 14;11(10):uhae235. doi: 10.1093/hr/uhae235 (PMC11489597; doi:10.1093/hr/uhae235)
Supplement: Web_Material_uhae235 [file web_material_uhae235.zip › Figure S4.docx]

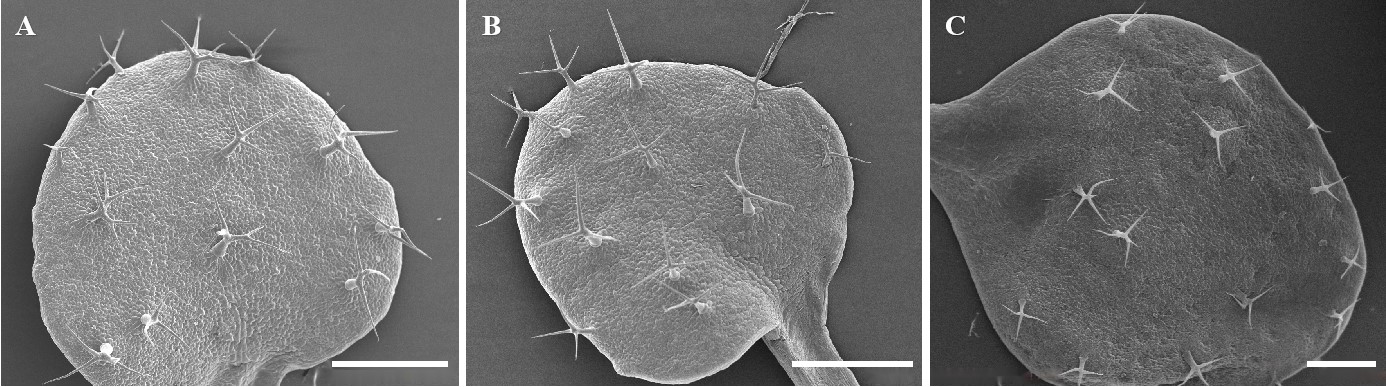


**Figure S4 SEM observation of *Arabidopsis* trichomes**

1. Wild-type; (B) T-DNA mutant of C-type LecRLK in *Arabidopsis*; (C) *oxCsTs* in *Arabidopsis*; Scale bars are 500μM.
