# Supplementary material for: CsTs, a C-type lectin receptor-like kinase, regulates the development trichome development and cuticle metabolism in cucumber (Cucumis sativus)
Source: Hortic Res. 2024 Aug 14;11(10):uhae235. doi: 10.1093/hr/uhae235 (PMC11489597; doi:10.1093/hr/uhae235)
Supplement: Web_Material_uhae235 [file web_material_uhae235.zip › Figure S6.docx]

**Figure S6 Schematic diagram of molecular regulation pathway for cucumber trichome development by Ts**

Interactor

Direct interaction

Epistatic regulation

Positive effect

Negative effect

Extracellular signal

Auxin signal


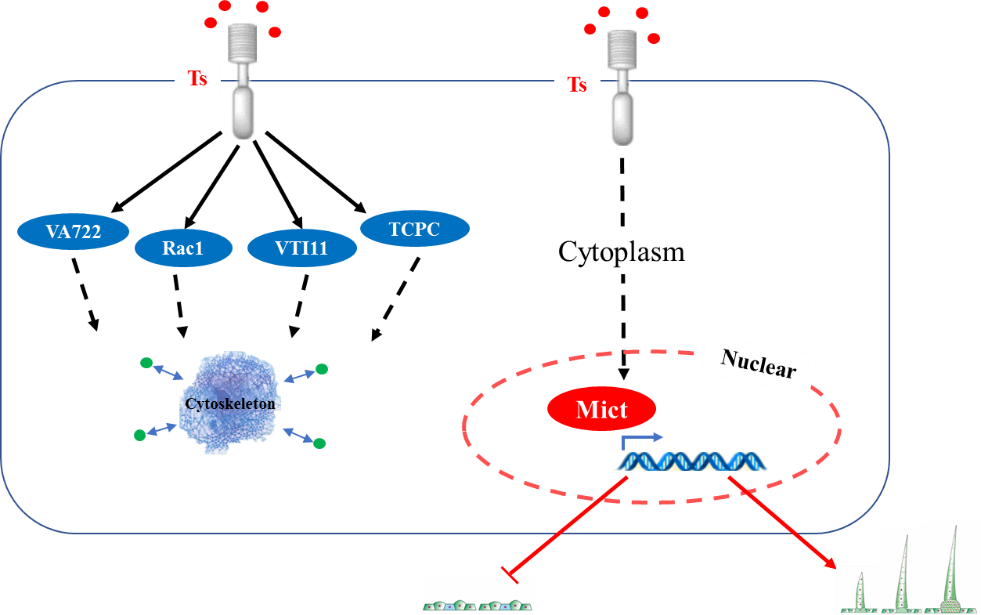


**Cytomembrane**
