# Supplementary figures and images for: CsTs, a C-type lectin receptor-like kinase, regulates the development trichome development and cuticle metabolism in cucumber (Cucumis sativus)
Source: Hortic Res. 2024 Aug 14;11(10):uhae235. doi: 10.1093/hr/uhae235 (PMC11489597; doi:10.1093/hr/uhae235)

**Figure S5 BiFC analysis between CsTs and CsMict proteins of cucumber**

Scale bars are 50μM.


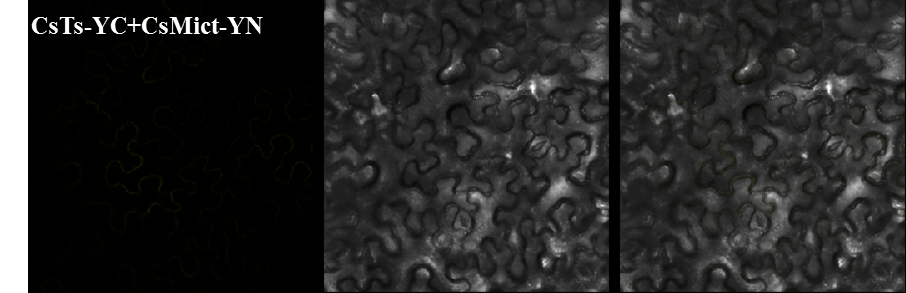

Supplement: Web_Material_uhae235 [file web_material_uhae235.zip › Figure S5.docx]
